# Technetium and blood extravasation before gammagraphy: a case report

**DOI:** 10.1186/1757-1626-2-141

**Published:** 2009-02-22

**Authors:** Sergio Vano-Galvan, Cristina Rodriguez-Rey, Eliseo Vano-Galvan, Pedro Jaén

**Affiliations:** 1 Dermatology Service, Ramon y Cajal Hospital, University of Alcala, Madrid, Spain; 2 Nuclear Medicine Service, San Carlos Hospital, Madrid, Spain; 3 Radiology Service, San Carlos Hospital, Madrid, Spain

## Abstract

**Background:**

An 80-year-old woman presented with an abrupt onset of asymptomatic black-to-purple discoloration of her right limb that appeared just before a thyroid gammagraphy. No antecedent of trauma was found.

**Case presentation:**

On questioning, patient admitted she did not cooperate during intravenous administration of Tc-99m-pertechnetate. Based on the abrupt onset of lesions and the absence of cooperation during intravenous administration of radiotracer, a suspect diagnosis of technetium and blood extravasation was made. This entity was confirmed in basis of evolution, with a spontaneous resolution of lesions within the next 15 days with no complications.

**Conclusion:**

Patient and physicians must be reassured because of the non-vesicant property of technetium. Recovery of this entity is spontaneous, and no treatment is needed.

## Case presentation

An 80-year-old woman presented with an abrupt onset of asymptomatic black-to-purple discoloration of her right limb that appeared just before a thyroid gammagraphy. No antecedent of trauma was found. Her medical history consisted on hypertension, hyperthyroidism and depression. On questioning, patient admitted she did not cooperate during intravenous administration of Tc-99m-pertechnetate.

**Figure 1:**
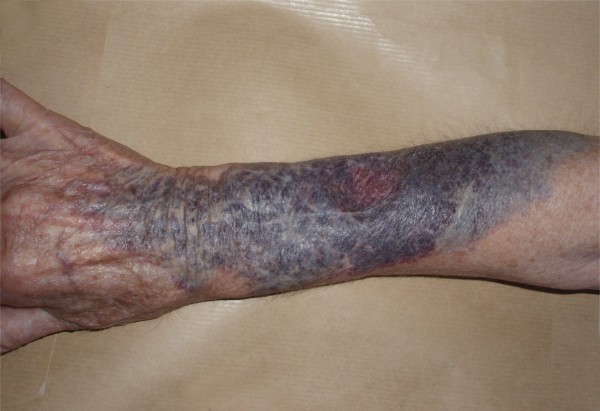
**Violaceous soft plaque located on the right arm that appeared after intravenous administration of Tc-99m-pertechnetate**.

Dermatological examination showed an extensive black-to-purple soft plaque located on her distal right upper extremity, with a central depressed reddish area that coincided with peripheral intravenous access (Figure. 1). The remainder of physical examination was within normal limits. Laboratory evaluation included a complete blood cell count and a prothrombin and partial thromboplastin time, all of which were normal.

Based on the abrupt onset of lesions and the absence of cooperation during intravenous administration of radiotracer, a suspect diagnosis of technetium and blood extravasation was made. This entity was confirmed in basis of evolution, with a spontaneous resolution of lesions within the next 15 days with no complications.

## Discussion

Soft tissue extravasation of radiotracer and blood after intravenous administration is a self-limited condition characterized by the appearance of a purple plaque around the area of peripheral intravenous access. It may result in imaging artefacts [1], so nuclear imaging procedure should be repeated later on.

Recognizing radiotracer and blood extravasation is important to avoid misdiagnoses such as skin necrosis or irritant reactions like those induced by chemotherapy [2], in which the severity of tissue injury is dependent on the type and concentration of the chemotherapeutic agent and the quantity injected. Cytotoxic agents may be classified as irritants or vesicants. Irritants are drugs that can cause an inflammatory reaction, aching, swelling, pain or phlebitis at the injection site or along the vein. Vesicants are drugs that may cause severe and lasting tissue injury and necrosis. Symptoms may arise immediately after extravasation or appear after several days or weeks. In case of a significant extravasation, necrosis, eschar formation and ulceration with involvement of underlying tissues may occur [3].

Patient and physicians must be reassured because of the non-vesicant property of technetium. Recovery of this entity is spontaneous, and no treatment is needed.

## Consent

Written informed consent was obtained from the patient for publication of this case report.

## Competing interests

The authors declare that they have no competing interests.

## Authors' contributions

SV-G wrote the initial draft of and helped revise the manuscript

CR-R and EV-G obtained consent from the patients and helped revise the manuscript

PJ assisted with manuscript revision. All authors read and approved the final manuscript
